# Biofeedback therapy using Cerebri for the prevention of migraine attacks in adults with episodic migraine (BioCer): a randomized, wait-list controlled trial – the study protocol

**DOI:** 10.12688/f1000research.149807.2

**Published:** 2025-08-29

**Authors:** Amalie Christine Poole, Anker Stubberud, Melanie Simpson, Lise Øie, Einar Tobias Vassbø Skalstad, Marte-Helene Bjørk, Espen Saxhaug Kristoffersen, Kjersti Grøtta Vetvik, Alexander Olsen, Iben Cornelia Keim Larsen, Mattias Linde, Erling Andreas Tronvik, Tore Wergeland Meisingset

**Affiliations:** 1 Norwegian Centre for Headache Research (NorHead), Norwegian University of Science and Technology, Trondheim, Norway; 2 Department of Neurology and Neurophysiology, St. Olavs hospital, Trondheim University Hospital, Trondheim, Norway; 3 Department of Neuromedicine and Movement Sciences, Faculty of Medicine and Health Sciences, Norwegian University of Science and Technology, Trondheim, Norway; 4 Department of Public Health and Nursing, Faculty of Medicine and Health Sciences, Norwegian University of Science and Technology, Trondheim, Norway; 5 Department of Clinical Medicine, University of Bergen, Bergen, Norway; 6 Department of Neurology, Haukeland University Hospital, Bergen, Norway; 7 Department of Neurology, Akershus University Hospital, Lørenskog, Norway; 8 Department of General Practice, Institute of Health and Society, University of Oslo, Oslo, Norway; 9 Department of Psychology, Norwegian University of Science and Technology, Trondheim, Norway; 10 Clinic of Rehabilitation, St. Olavs hospital, Trondheim University Hospital, Trondheim, Norway; 11 Department of Neurology, University Hospital of North Norway, Tromsø, Norway; 12 Regional Migraine Unit, Sahlgrenska University Hospital, Gothenburg, Sweden

**Keywords:** mHealth, biofeedback, behavioural therapy, headache, migraine, neurology

## Abstract

**Introduction:**

Biofeedback is a non-pharmacological treatment option valued for its minimal risk of adverse events and offers a safe alternative for individuals seeking preventive care for migraine. Despite level A evidence for migraine prevention, biofeedback treatment is still unavailable to most patients. We developed a novel medical device (Cerebri) for multimodal biofeedback treatment that omits the need for healthcare personnel involvement. Cerebri consists of a smartphone application (app) and two wireless sensors. Unique in its approach, the Cerebri app seamlessly integrates three biofeedback modalities – heart rate variability, temperature, and electromyography – making it a comprehensive, therapist-independent solution for non-pharmacological migraine management.

**Methods:**

Biofeedback therapy using Cerebri for the prevention of migraine attacks in adults with episodic migraine (The BioCer study) is an open-label, randomized, waitlist-controlled, multicenter trial. This study investigates the safety and efficacy of daily home-based biofeedback sessions using the Cerebri device. A total of 286 participants will be randomized to either a 12-week intervention arm or waitlist control arm. The primary outcome is the change in the mean number of migraine days from baseline to the last 28-day period during the treatment phase in the treatment group compared with the control group. The primary outcome variable is prospectively collected through daily eDiary entries. A limitation is the inability to conduct a sham-controlled trial of biofeedback.

**Ethics and Dissemination:**

Approval from the ethics committee and competent authorities was obtained prior to study initiation. Participation is voluntary and informed and written consent is obtained prior to inclusion. The results of this trial will be published in peer-reviewed international medical journals and communicated to patients and healthcare personnel through the relevant channels.

**Trial registration numbers:**

EUDAMED: CIV-NO-22-08-040446

REK (Regional Committees for Medical and Health Research Ethics): 502734 Date of approval 2022-10-14

Trial registration: NCT05616741, 2022-11-15, https://clinicaltrials.gov/study/NCT05616741

## Introduction

Migraine is the second most disabling disorder worldwide and incurs enormous societal costs, estimated to exceed €50 billion per year in the EU alone. ^1^^,^^2^ Frequent migraine attacks warrant preventive treatment. A multitude of preventive pharmacological options are available; however, there are several barriers to effective treatment. For instance, one study reported that over two-thirds of patients discontinued their prophylactic migraine medication after six months, and more than 80% after a year, due to either a lack of efficacy or adverse reactions. ^3^

Several non-pharmacological behavioral interventions for migraine, such as biofeedback, have been highlighted as non-invasive prophylactic treatment regimens with a low risk of negative side effects, which can be an alternative or adjunct to pharmacological therapies. Biofeedback combined with relaxation training is listed with Grade A level evidence for migraine prevention in the 2000 guidelines from the American Academy of Neurology. ^4^ This has been continued in the updated 2021 American Headache Society consensus statement, which also suggests mobile health (mHealth) application-based approaches for delivering biobehavioural treatments to increase patient access and participation. ^5^

Biofeedback utilizes technologies and equipment to monitor physiological processes that are usually considered involuntary and modulated without conscious awareness. Presented with real-time feedback on these processes, users can learn to exercise control over them, which in turn has a beneficial effect on the migraine symptom burden. Despite its effectiveness, biofeedback has limited accessibility. The high financial costs of having a therapist instructing the procedure, necessary equipment, and patient travelling costs are likely to contribute to this. To our knowledge, no therapist-independent migraine biofeedback treatment with proven efficacy is currently available. ^6^

To address this gap, a multimodal biofeedback system, the Cerebri system, has been developed for therapist-independent home-based use. Unique to this product, the Cerebri application (app) seamlessly integrates three biofeedback modalities: heart rate variability (HRV), temperature, and electromyography (EMG), making it a comprehensive, home-based solution for non-pharmacological migraine management. The device is a product of a thorough development process, including a hardware feasibility study ^7^ and three development studies on progenitor versions of the system with short test periods. ^8^^–^^10^ In addition, prior to the initiation of this trial, a feasibility study with 3-month follow-up was performed. ^11^

## Protocol

### Study objectives and endpoints

The objective of this study is to investigate the efficacy and safety of a 12-week daily use of the Cerebri biofeedback device as compared to a waitlist control in adults with episodic migraine.

Primary efficacy endpoint

The primary endpoint is the change in the mean number of migraine days from baseline to the last 28-day period (week 9–12) during the treatment phase in the treatment group compared to the wait-list control group.

Secondary efficacy endpoints

The key secondary endpoint analyses are changes in mean number of migraine days in week 1–4 and week 5–8, change in mean migraine intensity, proportion of 30% responders, change in acute migraine drug treatment use, and change in subject-reported headache-related disability. Exploratory and descriptive endpoints include changes in weekly migraine days, mean headache days per 28-day period, eDiary and biofeedback session adherence, and mean Patient Global Impression of Change (PGI-C) scores. Additionally, changes in the mean number of migraine days during the extended biofeedback period will be assessed.

Safety endpoints

Description of the frequency and severity of treatment-emergent adverse events (AEs), adverse device effects (ADEs), serious adverse device effects (SADEs), and unexpected serious adverse side effects (USADEs) is included. In the case of pregnant participants, at inclusion or acknowledged during participation, no additional monitoring of maternal or fetal health is instituted. In such cases, participants are asked if they agree to be followed up until the end of pregnancy to collect data on any pregnancy complications and fetal outcome data.

## Methods and analysis

### Study design

This is an open-label, randomized, waitlist-controlled, multicenter trial. The study design follows the standard for clinical investigation of medical devices for human subjects – good clinical practice (ISO 14155:2020) – and the relevant trial guidelines of the International Headache Society. ^12^ Reporting follows the CONSORT guidelines.

**Figure 1.**
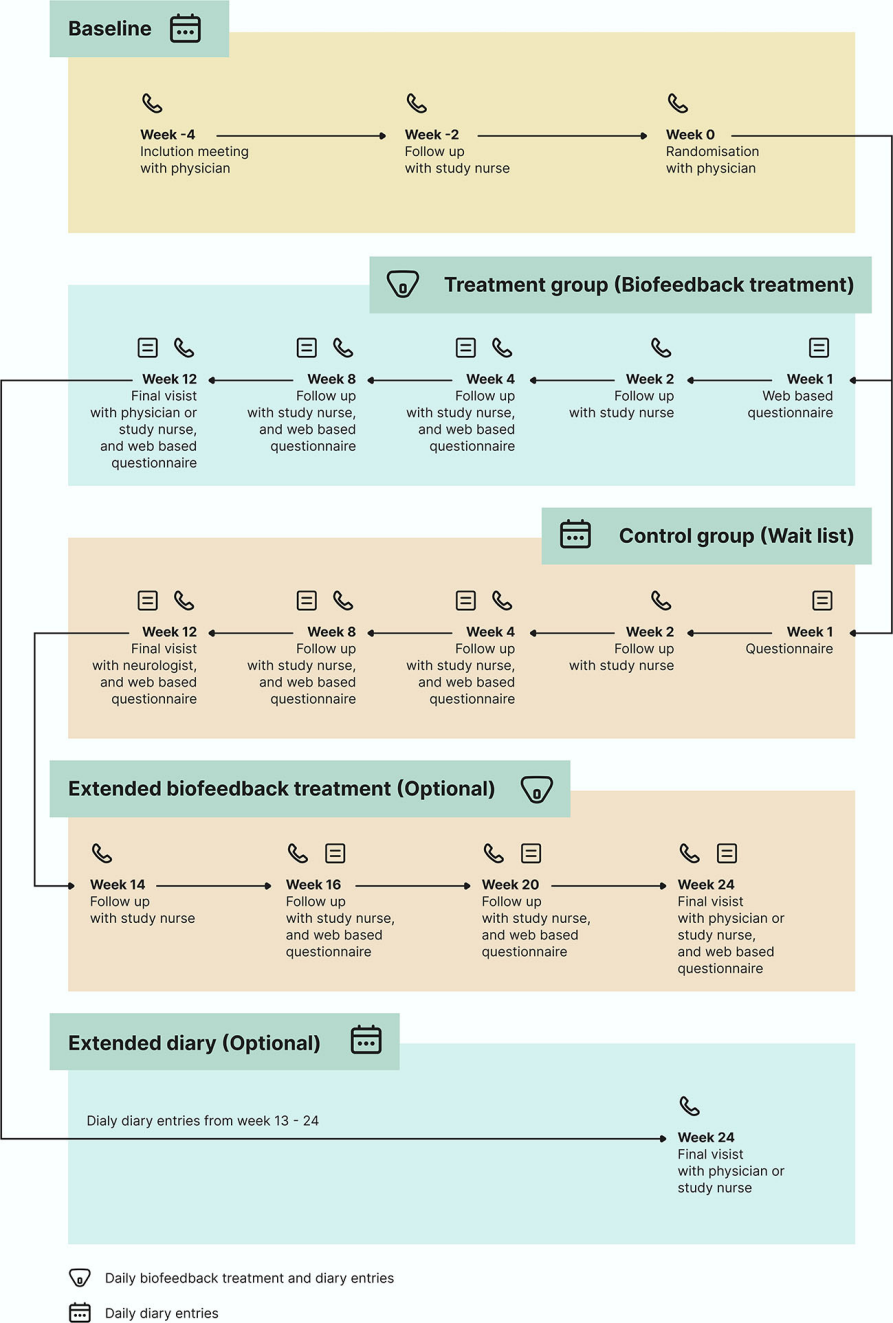
Flow chart of the BioCer study.

The total duration of study participation is 16 weeks, with the possibility of a 12-week extension period ( Figure 1). After the screening visit, if eligible, the participants are assigned to a baseline period of a minimum of four weeks in accordance with the guidelines. ^12^ During the baseline period, participants maintain a daily headache diary in the Cerebri smartphone app. Upon completion of the baseline period, the participants were rescreened for eligibility prior to randomization. This design, where the baseline period also serves as a screening period, enables rescreening based on eDiary entries. This is the recommended approach for enrolling RCTs on episodic migraine. ^12^ Participants deemed ineligible at the point of rescreening, or who withdraw from or are lost to follow-up during the baseline period, are categorized as screening failures and are excluded from further participation.

Eligible participants are randomly allocated to the treatment or waitlist groups. The treatment group receives the medical device by postal service, and a treatment period of 12 weeks is initiated on the first day of completing a biofeedback session. Participants in the waitlist group complete daily eDiary entries for 12 weeks from the day of randomization.

Both arms are invited to participate for a 12-week extension period. Participants randomly allocated to the waiting list are offered the possibility of entering a 12-week extension phase with biofeedback treatment. The 12-week extension period is initiated on the first day of the biofeedback training. The participants randomized to the intervention group are invited to a 12-week extension period after completing their 12-week biofeedback treatment, in which they continue with daily eDairy only.

Participants will not have access to the intervention at the end of the study. Beyond the voluntary 12-week extension period, there will be no long-term follow-up on safety and survival status, except in cases of pregnancy.

### Randomization and stratification

Random group allocation occurs a minimum of four weeks after inclusion and after rescreening. If eligible, permuted block randomization is performed. Randomization is stratified on the adjunct use of preventive medication.

### Design of control group

Blinding/masking is not possible owing to the nature of the intervention in question and is thus not performed.

### Recruitment

This trial recruits participants from all over Norway, with inclusion sites covering all health regions. The investigational sites are the neurological departments at St. Olavs Hospital, Trondheim University Hospital, Akershus University Hospital, Haukeland University Hospital, and University Hospital of North Norway. Visits 1 (baseline inclusion) and 2 (rescreening and randomization) are performed by medical doctors with headache expertise. All visits are conducted remotely via video or telephone. Information about the trial and contact information to indicate interest in participation is disseminated through relevant institution webpages and social media platforms. Interested parties may contact the study site directly via email or may be referred. Study personnel are prohibited from enrolling patients for whom they were primarily responsible for treatment and/or follow-up.

### Eligibility criteria

A full list of the inclusion and exclusion criteria is listed in Table 1. Eligibility is assessed at visits 1 and 2 ( Figure 1), and participants must comply with all inclusion criteria and none of the exclusion criteria to proceed to randomization. Participants are allowed to use one preventive migraine medication if they are stable upon study entry and remain unaltered during study participation. In the case of onabotulinum toxin A injections following the PREEMT injection paradigm, a stable treatment regimen is operationalised as at least three consecutive treatments prior to inclusion. All preventive treatments in use was initiated as part of previous management prior to study entry, and this clinical follow up was continued during trial participation. For other prophylactic treatments, a duration of three months or five half-lives, whichever is longer, of unaltered use is considered stable.

**Table 1.** Eligibility criteria for participation in the BioCer trial.

Inclusion criteria:	Exclusion criteria:
1. 18 years of age or older. 2. Episodic migraines with or without aura diagnosed by a neurologist/physician per International Classification of Headache Disorders 3 ^rd^ edition (ICHD-3). 3. History of at least 4 and up to 14 days of migraines per 28-day period in the 3 months prior to screening (as recalled by the subject). This frequency must be confirmed in the headache diary before randomization to treatment or wait-list control. 4. At least three months of experience with smartphone and access to an iOS or Android phone at home. 5. Capable of giving signed informed consent with includes compliance with the requirements and restrictions listed in the informed consent form (ICF) and in this protocol. 6. Onset of migraine before age 50 years.	7. More than 14 days of headache (all types) per 28-day period. 8. Subjects diagnosed with trigeminal autonomic cephalalgias and neuralgias. 9. Subjects with secondary headache conditions. 10. Subjects with pathologies that inhibit use of the device according to the instructions for use (e.g. blindness, deafness). 11. Use of non-pharmacological preventive treatment (meditation, physical therapy, psychotherapy as a headache treatment, acupuncture etc.) with the exception of stable treatment for other indications than migraine. 12. Use of concurrent migraine preventive medication, with the exception of stable dose ( ≥ 3 months) monotherapy of migraine preventive medication. 13. Subjects taking opioids ( ≥ 3 days per month) or barbiturates at the time of screening. 14. Subject participates in another clinical investigation or has participated in CER-MIG-1. 15. Alcohol overuse or illicit drug use. 16. Subject who is unlikely to follow Clinical Investigation Plan or where treatment seems futile in the opinion of the investigator or have demonstrated an inability to sufficiently adhere to headache diary entries (<70%).

### Ethics

This study is conducted in accordance with the principles of ISO 14155:2020 (Clinical Investigation of Medical Devices in Human Subjects), Good Clinical practice, and the Guidelines of the International Headache Society. The study is approved by the Regional Committees for Medical Research Ethics (REK, approval number 502734. Date of approval 2022-10-14) and the Norwegian Medical Product Agency (NOMA) (reference number 22/17254-5. Date of approval 2022-09-01) prior to initiation. Substantial protocol amendments will be reviewed by competent authorities before implementation. Data protection follows the GDPR. We adhere to the Declaration of Helsinki.

To ensure that all subjects have similar opportunities to benefit from enrolment, participants randomized to the waitlist group are offered treatment following the conclusion of the waitlist period. Consent is collected by the investigator conducting the screening visit. Study information is sent in advance of visit 1 by email, scripted study information is provided orally, and any questions are addressed during visit 1 prior to signing to ensure that consent is obtained in all cases. Consent is collected by a digital signature via a national electronic data entry application («Helsenorge»). Study personnel are prohibited from recruiting patients for whom they are directly responsible for treatment.

### Study intervention

Investigational medical device

**Figure 2.**
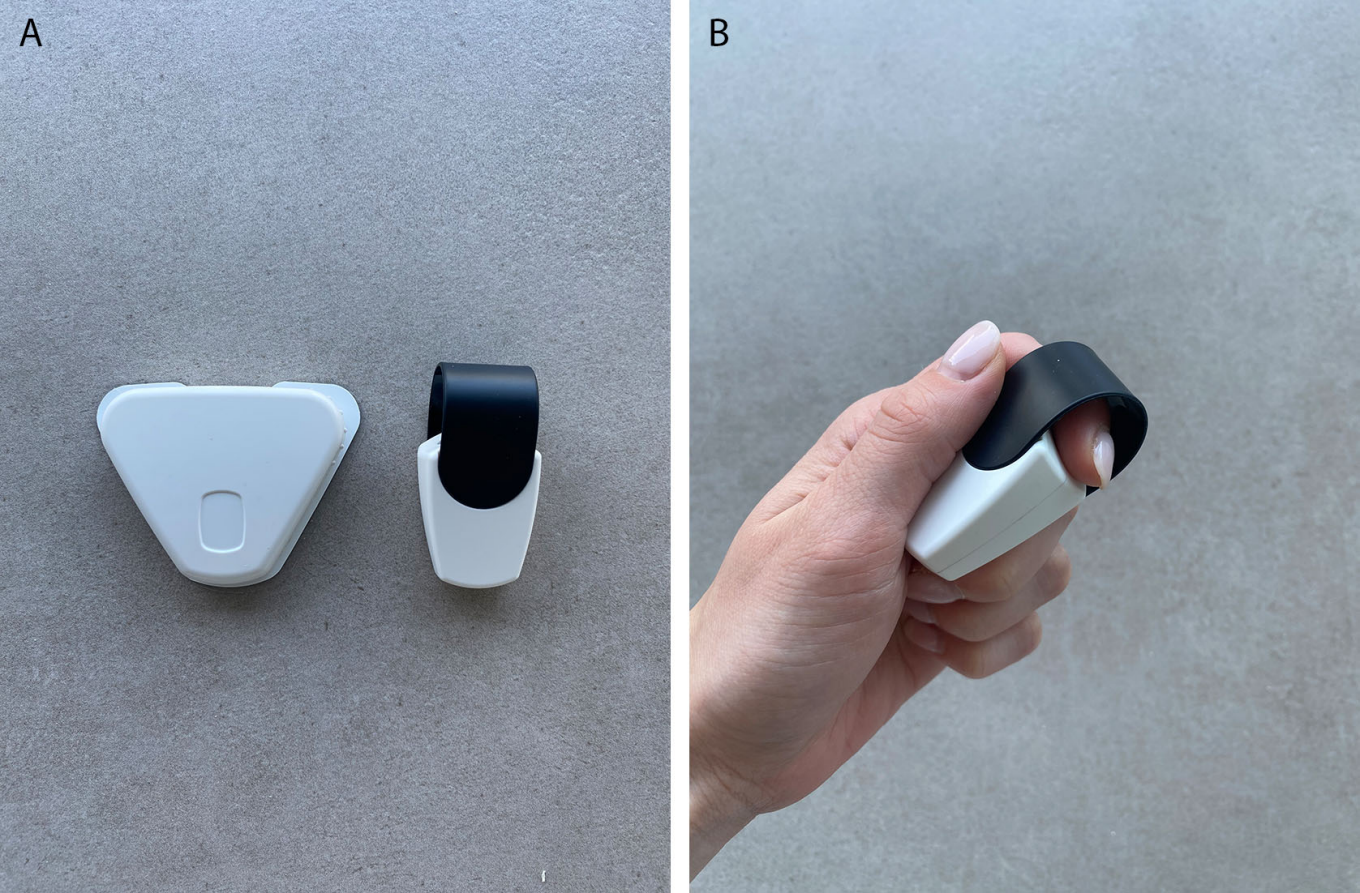
A, B: The investigational medical device Cerebri finger sensor and EMG sensor.

**Figure 3.**
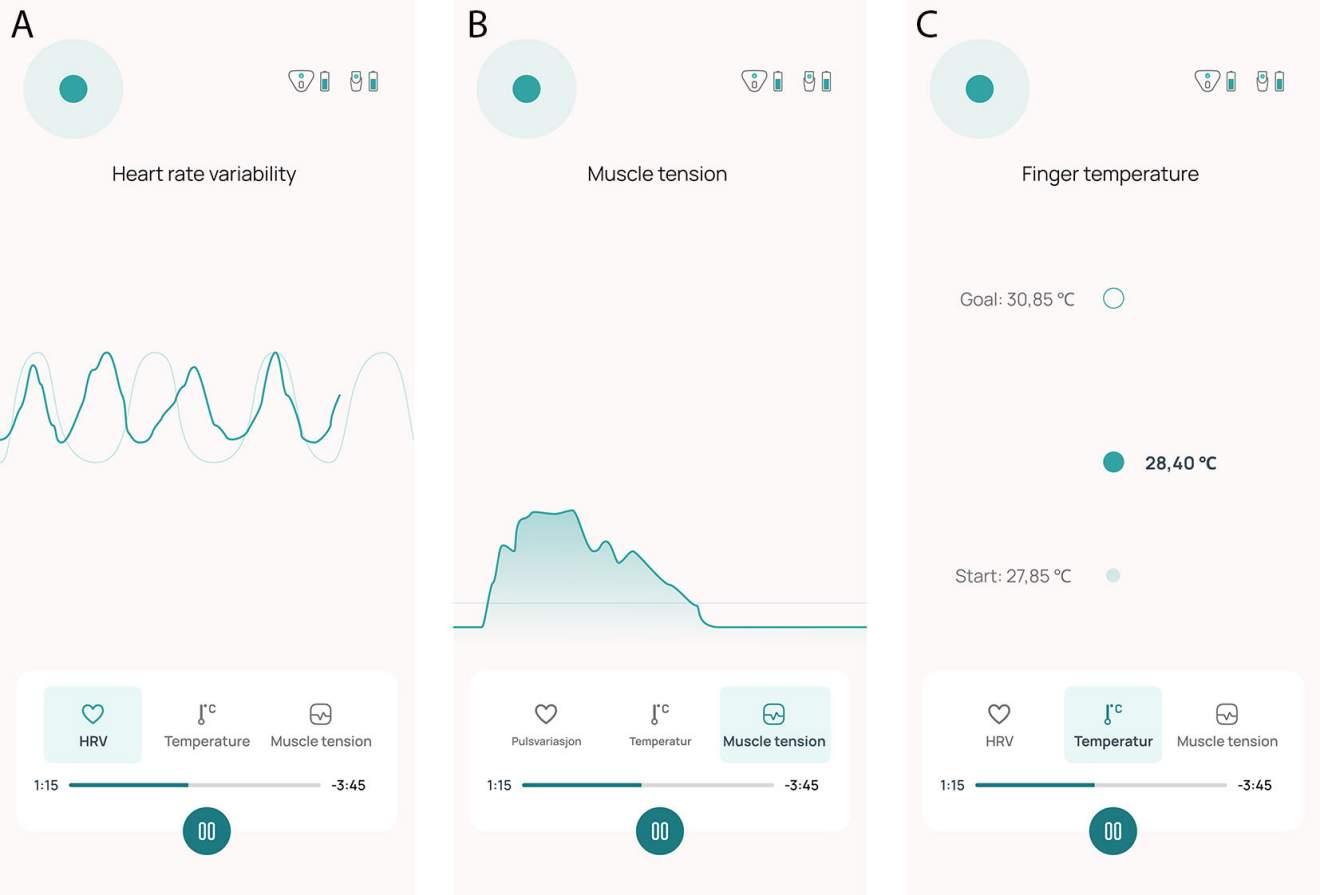
Screen shots of the biofeedback treatment in the Cerebri mobile application, showing the HRV (A), EMG (B) and temperature (C) modality interface.

The medical device Cerebri consists of a smartphone app and a set of two wireless non-invasive sensors. Both sensors are skin-contact-dependent. One sensor is attached to the index finger and measures HRV and peripheral skin temperature. The second sensor is attached to the skin over the upper trapezius muscle fibers using adhesive electrodes and measures muscle tension as surface electromyographic voltage ( Figure 2A and 2B). The sensors transmit the measurements to the user’s smartphone by Bluetooth technology and display them in real time to the user. Participants receive the sensors by mail together with a brief written onboarding guide. All training is delivered in a standardised, app-based format without individualised or therapist-led instruction. The app guides users through the first three biofeedback sessions, and provides a library with textual instructions on exercises that the user can choose to use in their biofeedback training. By following instructions for suggested exercises displayed in the Cerebri smartphone app, the subject trains on influencing the measured parameters in the desired direction during each biofeedback session. Figure 3A-C illustrate how visualization of the physiological measures is displayed on their smartphone. Exercises to facilitate successful manipulation of the physiological parameters include breathing techniques, progressive muscle relaxation, and awareness training. A visual breathing pacer to support paced breathing is available in the mobile app to the user during the entire treatment session. It is desirable to increase HRV and peripheral skin temperature and lower neck muscle tension. Participants are instructed to sit comfortably in a quiet room during treatment. Throughout the intervention period, participants are required to complete a 10-minute biofeedback session at least once daily at a time of their choosing. They have the option to perform as many as six sessions each day.

### Data collection

eDiary and biofeedback

**Figure 4.**
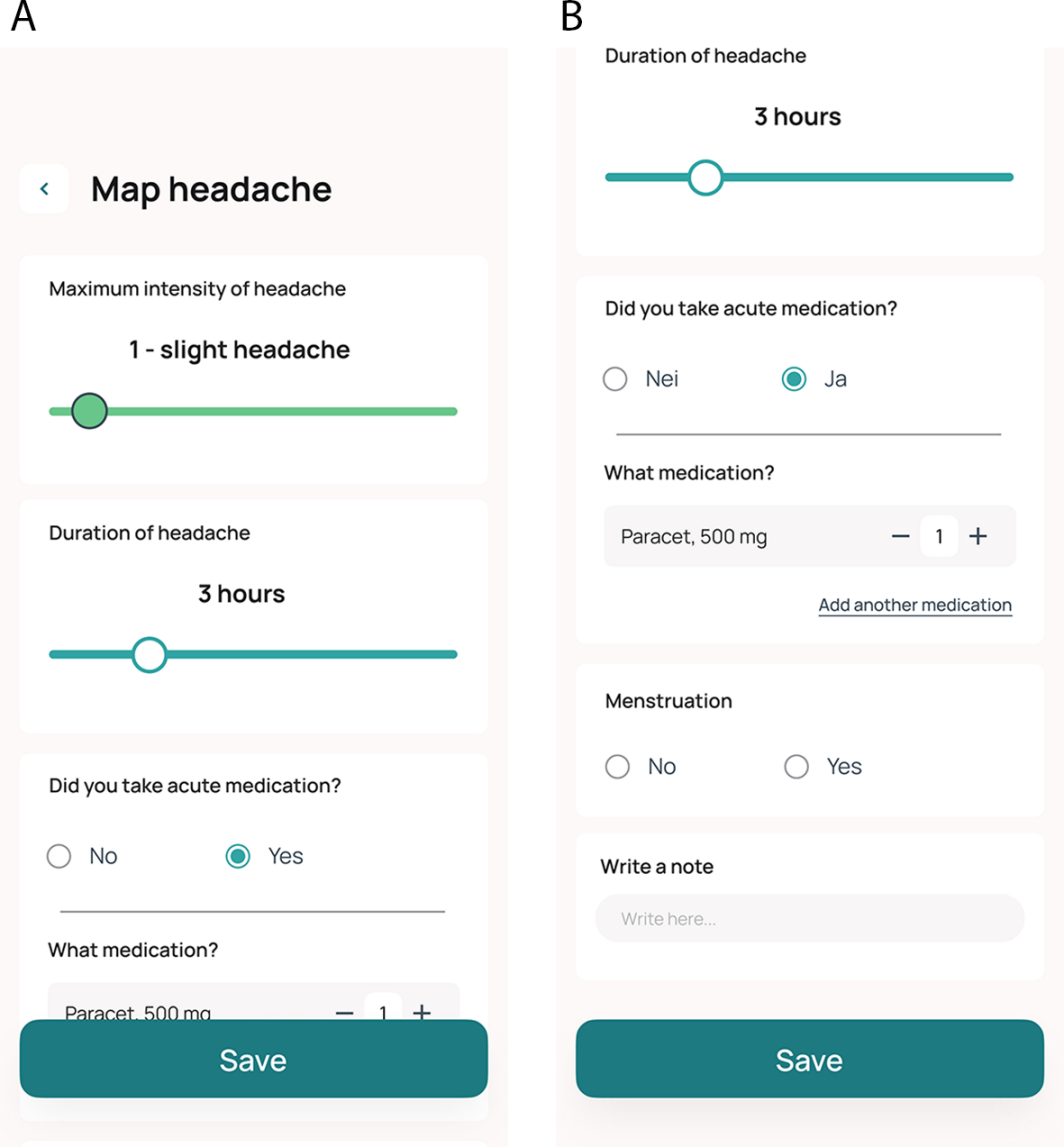
A and B: Screen shots of the eDiary in the Cerebri mobile application.

Data on headache is collected prospectively as a patient-reported outcome measure in an eDiary integrated into the Cerebri smartphone app. Daily entries are encouraged, but the app allows the entry of registrations from the two previous calendar days. The app includes daily reminders to promote adherence. The tool is designed such that when participants report headaches, they are prompted to answer additional questions. Data collected in the diary include the estimated duration of headache, intensity of headache using an 11-point scale (0-10), and the use (type, dose, and number of administrations) of acute medications (triptan, paracetamol, NSAIDs, opioids, other), and information on whether acute medications relieved headache ( Table 2 and Figure 4A and 4B). Participants are followed up with telephone calls at week 2, week 4 and week 8 to promote adherence ( Figure 1).

**Table 2.** Overview of the eDiary variables used for the mobile application.

Variable	Data input
Headache	Yes/No
Headache recognized as migraine	Yes/No
Intensity of headache	1-10
Duration of headache	0.5 to 24 hours
Acute medication	Yes/No
Type of medication	Medication name and dosage
Did the acute medication alleviate the headache	Yes/No
Menstruation	Yes/No

A web-based dashboard solution enables study personnel to access a real-time overview of adherence to eDiary and biofeedback treatment. Thus, study personnel can identify and reach out to participants with low adherence. In the case of technical issues, a support service provided by the sponsor is available. The dashboard also includes information regarding the migraine/headache status on a given day and acute medication use. This information will be accessible to study personnel in the baseline period for rescreening purposes. Post randomization, only the adherence data is available.

Questionnaires

Subjects are asked to complete validated questionnaires at the end of each 28-day period to measure the headache-related impact on the quality of life and their impression of treatment efficacy. The questionnaires used in this trial are the Migraine-Specific Quality-of-Life Questionnaire (MSQ) v2.1 ^13^ and the Patient Global Impression of Change (PGI-C). ^14^ The MSQ is copyrighted and registered with the United States Library of Congress by Glaxo Wellcome, Inc. A written permission for use is obtained prior to use.

Electronic patient record

Patient records are the source of information regarding medical history, current medications in use, and previous trials of prophylactic migraine treatment acquired during visit 1. Alterations in health status, including treatment-emergent adverse events during trial participation are registered in the medical records.

Study-specific electronic case report form (eCRF)

An eCRF applying the WebCRF3 software (version 3) ( https://webcrf3.medisin.ntnu.no) was developed for this study. The webCRF software is provided and maintained by the Unit for Applied Clinical Research (AKF) at the Faculty of Medicine and Health Sciences, NTNU. Data collected during study visits, telephone consultations, and safety information are entered. Data are stored in a pseudo-anonymous manner, and each study participant is identified using a unique study identification (ID) number. An open-source alternative OpenClinica ( https://www.openclinica.com/get-free-community-edition-software) is able to perform an equivalent function as WebCRF3.

Study monitoring

No data-monitoring committee is appointed for this study. An independent Data Monitor will be used for review of the study in accordance with a Monitoring Plan.

Data management

Data management is guided by the data management plan. Data validation and verification of a minimum of 10% of participants will be conducted before data lock. Data lock can be performed when the last participant ends its 12 week post-randomization period, that is, while the participants are still in an extension period.

### Statistical analysis

Research hypothesis

The primary endpoint is to investigate the change in the mean number of migraine days from baseline to the last 28-day period in the treatment group compared with the wait-list control group. Thus, the null hypothesis (H0) and alternative hypothesis (H1) to be tested in relation to the primary objective are as follows.

• H0: The change in the mean number of migraine days from the 28-day baseline to the last 28-day period in the treatment group is equal to the change in the mean number of migraine days from baseline to the last 28-day period in the wait-list control group.

vs.

• H1: The change in the mean number of migraine days from the 28-day baseline to the last 28-day period in the treatment group is not equal to the change in the mean number of migraine days from baseline to the last 28-day period in the wait-list control group.

Sample size

The sample size calculation is based on an assumption of the effect size derived from a relevant meta-analysis of the efficacy of biofeedback in treating migraine ^15^ and a randomized pilot study. ^9^ Provided an effect size of d = 0.4, a significance level alpha of 0.05 (two-sided), power 0.8, and a dropout rate of 30% in the post-randomization period, the required sample size of this study was estimated to 286 participants. The anticipation of high dropout rates in this study is justified in the significant challenge of maintaining participant adherence observed in home-based mHealth studies. ^9^^,^^16^

Protocol deviations

Participants with incomplete or missing headache diary entries in more than 30% of days in the post-randomization period, participants with more than 30% missing or incomplete biofeedback sessions in the treatment arm, and participants initiating new or altering the use of their established preventive headache medications during the trial, are considered protocol deviators.

Statistical analysis plan

The statistical analysis plan (SAP) will include a technical and detailed description of statistical analysis. Statistical analyses will be conducted by an independent statistician. The analysis will follow a predefined order, where the primary efficacy endpoint is analyzed first. The trial statistician will analyze the efficacy outcomes without knowledge of treatment allocation by using a dataset with study arm allocation coded as groups A and B.

Data analysis set

For the efficacy analysis, the full analysis set includes all randomized participants and constitutes the intention-to-treat (ITT) population. The per protocol (PP) population includes all randomized participants without protocol deviations.

Analysis dataset 1 is defined as the data from the 16-week period for all randomized participants and does not include extension period data. The 12-week post-randomization period in the biofeedback arm starts on the date of the first biofeedback session. Data lock for the evaluation of efficacy endpoints can be performed upon the completion of this dataset.

The safety data set includes all participants exposed to the biofeedback device.

Primary endpoint analysis

Analysis of the study endpoints will be performed in the ITT (all randomized subjects) and PP populations.

The primary endpoint variable is migraine days. The operational definition used for the analysis will be prespecified in the SAP.

The planned analysis strategy involves a mixed logistic regression model which will include all available daily eDiary entries. The mixed logistic regression model will be used to estimate the difference in the change from baseline in the mean number of migraine days per 28-day period between the treatment and waitlist groups. The model will include the presence or absence of migraine on each day from the baseline and 12-weeks post randomization periods as a dependent binary variable, treatment allocation, time period (baseline, week 1-4, week 5-8 and week 9-12) and an interaction term between treatment allocation and post-randomisation time-periods are included as categorical fixed effects, together with the stratification variable (concomitant preventive medication use). The patient ID will be included as a random effect. The site of inclusion will also be assessed as a potential random effect prior to the unblinding of the statistician to the treatment allocation. The model does not require explicit imputation, as mixed models yield unbiased estimates under the assumption that data are missing at random. This assumption will be assessed and sensitivity analyses considered if warranted.

The primary effect estimate will be the model-based estimates of the difference in change from baseline in mean monthly migraine days between the treatment and waitlist groups, presented with a 95% confidence interval.

Baseline variables that are considered to be highly predictive of outcomes will be discussed and identified *a priori* and described in the SAP. These variables will be included in multivariate analysis. The SAP will also detail the analysis strategies for the secondary outcomes. Post hoc stratified analyses by sex (women vs. men) and assessment of sex interaction will be conducted.

The statistical analysis plan includes predefined sensitivity analyses, restricting the primary analysis to the per-protocol set and, if feasible, subgroup analyses according to adherence status. Specifically:

• Participants in the interventional group with >90% biofeedback adherence in all three 28-day periods will be compared with the remaining participants in the interventional arm with lower adherence.
• Participants in the interventional group with >90% adherence during weeks 1–4 will be compared with the remaining participants in the interventional arm with lower adherence in that same period.

Safety data

Safety objectives will be assessed by collecting data on treatment-emergent adverse events encountered during biofeedback training, including adverse events (AEs), serious adverse events (SAEs), adverse device effects (ADEs), serious adverse device effects (SADEs), and unanticipated SADEs (USADEs), and by evaluating the frequency and severity of these occurrences.

All data on treatment-emergent adverse events, ADEs, SADEs, and USADEs will be categorized and summarized. The frequency and severity of treatment-emergent AEs, ADEs, SADEs, and USADEs will be summarized for all enrolled participants and the entire duration of the intervention. Adverse events occurring more than once in individual participants will also be summarized and presented descriptively.

### Dissemination

The results of this trial will be published in peer-reviewed international scientific journals. The aim is for results to be made available and spread widely to the public, general practitioners, neurologists, and other health professionals who treat patients with headache. The results will be communicated to patients through relevant channels, such as social media, patient organizations, journals for popular science, and appropriate events and conferences.

### Study status

Study start 01.01.2023.

## Discussion

To the best of our knowledge, this is the first pivotal randomized controlled trial (RCT) with an adequate sample size to properly evaluate the efficacy of home-based biofeedback treatment in patients with episodic migraine. A 2022 systematic review of digital headache interventions identified only two RCTs evaluating biofeedback apps for migraine. ^17^ Both were pilot studies with small sample sizes and did not show statistically significant between-group differences in important headache related outcomes. ^9^^,^^16^

The uniqueness of Cerebri is that it combines three different biofeedback modalities, whereas traditional treatments typically use one parameter. ^15^^,^^18^ In addition, recent app-based solutions have applied one modality. ^16^^,^^19^^,^^20^ Different modalities may benefit different users; for example, some users might find it easier to influence their finger temperature, whereas others might prefer muscle tension as their main source of biofeedback. Different modalities also display different responses over time. Finger temperature increases slowly as the user relaxes and lowers their sympathetic tone, whereas muscle tension and HRV respond relatively quickly to activities such as neck shrugging and unpaced breathing, respectively. Together, these varying modalities target different pathomechanisms and potentially embrace more users as individuals often master different forms of autoregulation. The use and combination of these three different modalities have been assessed thoroughly in preliminary studies using a progenitor version of the Cerebri system. First, a feasibility study was conducted to confirm the precision of using surface EMG measured from the skin overlying the upper trapezius fibers to measure neck muscle tension and using a photoplethysmograph placed on the index finger to measure peripheral skin temperature. ^7^ Second, a usability study was conducted showing that it is possible to combine the modalities of neck muscle tension, finger temperature, and heart rate to tailor therapist-independent feedback to different users. ^8^ Finally, HRV was included as the third parameter instead of simple heart rate measurement, as it was demonstrated in a study from the US, ^16^ to be both feasible and acceptable for app-based biofeedback. The non-invasiveness of the Cerebri-system is advantageous in several ways. First, it enables the patients to safely perform the procedure themselves. Second, it means the risk profile is relatively low, with the most severe identified risks being electrical failure in the sensors or charger, skin contact-dependent allergic reactions from the sensor material, and risks related to information security and privacy. All the identified risks were met using an appropriate mitigation strategy. Third, a wider spectrum of patients qualifies for study inclusion; notably, women of childbearing potential who do not use highly effective contraceptives, as well as those who are pregnant or breastfeeding.

The BioCer trial is not blinded and employs a waitlist control. In a previous sham-controlled trial involving adolescents that tested an earlier version of the Cerebri system, the investigators encountered difficulties in developing a biofeedback sham that effectively imitated authentic biofeedback. ^9^ False feedback was easily detected by the user, compromising the integrity of the blinding protocol. Conversely, attempts to distort the biofeedback signal by introducing minor “errors” resulted in a sham signal that was “too similar” to true biofeedback, potentially inducing a treatment effect. In addition to the practical challenge of developing an appropriate sham signal, there are significant ethical concerns associated with providing false biofeedback to participants. These concerns include the potential exacerbation of migraines. Indeed, in theory, introducing a false biofeedback signal could lead to mislearning, in which subjects inadvertently adapt incorrect or maladaptive responses based on what they believe to be true biofeedback. Nevertheless, the absence of blinding is a limitation, as it prevents assessment of the placebo effect.

We believe that the effectiveness of Cerebri depends on achieving adequate user satisfaction and adherence to ensure optimal results. However, a recurrent challenge for smartphone-based biofeedback is its low adherence. A pilot study in adolescents of a progenitor version of Cerebri reported poor adherence, with a mere 40% of planned biofeedback sessions completed during weeks 5-8 of the study. ^8^ Similar adherence challenges have been reported for smartphone-based systems. In the feasibility study of the current Cerebri system proceeding this trial, biofeedback adherence was also low, declining from 80% in week 1-4 to 20% in week 9-12. ^11^ Several usability and software issues were identified and addressed before trial initiation.

To improve the usability of the Cerebri mobile app, additional data were added to the diary and visualized in graphs to provide users with better tools to keep track of their headaches and to compare headache data with biofeedback data directly. In addition, the app content was revised and instructions were improved based on reported use errors or misinterpretations. Based on user insights, the app interface during the biofeedback sessions was tailored to ensure that the biofeedback was performed correctly. Furthermore, the algorithm for calculating the HRV score was modified to compensate for drifting biofeedback scores resulting from frequency leakage from the low-to high-frequency domains of the HR wave. Some drifting still occurs, but it is deemed acceptable as the users are considered unable to observe this.

## Conclusion

Cerebri started as a research project in 2015, with a meta-analysis of the biofeedback literature on adolescents ^21^ and concept development on how to enable remote biofeedback for headache patients. The project utilized existing technology in the market, and as the concept developed, custom-made sensors and a more advanced biofeedback app interface were developed over the course of several years. Clinical testing of feasibility and usability continued to show positive feedback from the patients ^8^^,^^10^; hence, the project continued to validate the clinical effect of Cerebri. A feasibility study is important in validating the clinical study setup and defining the final iterations of the Cerebri user interface as the final preparation for this randomized controlled trial on Cerebri.

### Ethics

This study is conducted in accordance with the principles of ISO 14155:2020 (Clinical Investigation of Medical Devices in Human Subjects), Good Clinical practice, and the Guidelines of the International Headache Society. The study was approved by the Regional Committees for Medical Research Ethics (REK, approval number 502734. Date of approval 2022-10-14) and the Norwegian Medical Product Agency (NOMA) (reference number 22/17254-5. Date of approval 2022-09-01) prior to initiation. Substantial protocol amendments will be reviewed by competent authorities before implementation. Data protection follows the GDPR. We adhere to the Declaration of Helsinki.

To ensure that all subjects have similar opportunities to benefit from enrolment, participants randomized to the waitlist group are offered treatment following the conclusion of the waitlist period. Consent was collected by the investigator conducting the screening visit. Study information was sent in advance of visit 1 by email, scripted study information is provided orally, and any questions are addressed during visit 1 prior to signing to ensure that consent is obtained in all cases. Consent was collected by a digital signature via the national electronic data entry application («Helsenorge»). Study personnel are prohibited from recruiting patients for whom they were directly responsible for treatment.

## Authors information

EAT, AO, and AS conceived of the study. AS initiated the study design. TWM has the role of project leader and national coordinating investigator. MS and TWM wrote the statistical analysis plan and MS will conduct the primary statistical analysis. ETVS and ACP prepared the manuscript for publication. ACP, LRØ, MHB, KGV, ESK, IKL, and TWM are study investigators. All authors contributed to the refinement of the study protocol and approved the final manuscript.

## Data Availability

No data is associated with this article. Figshare: SPIRIT checklist for ‘Biofeedback therapy using Cerebri for the prevention of migraine attacks in adults with episodic migraine (BioCer): A randomized, wait-list controlled trial. https://doi.org/10.6084/m9.figshare.25480723.v1. The data are available under the terms of the Creative Commons Licence (CC BY 4.0).
