# Supplementary material for: Biofeedback therapy using Cerebri for the prevention of migraine attacks in adults with episodic migraine (BioCer): a randomized, wait-list controlled trial – the study protocol
Source: F1000Res. 2025 Aug 29;13:775. Originally published 2024 Jul 8. [Version 2] doi: 10.12688/f1000research.149807.2 (PMC12405843; doi:10.12688/f1000research.149807.2)
Supplement: Peer review history

# Reviewer report: Reviewer response for version 2

**DOI:** 10.5256/f1000research.187286.r410033

**Published:** 2025-09-02

**Authors:** Lanfranco Pellesi

**Affiliations:** 1 University of Southern Denmark, Odense, Denmark

I am satisfied with the current version of the manuscript.

Is the study design appropriate for the research question?

Yes

Is the rationale for, and objectives of, the study clearly described?

Yes

Are sufficient details of the methods provided to allow replication by others?

Yes

Are the datasets clearly presented in a useable and accessible format?

Not applicable

Reviewer Expertise:

Clinical trials in pain and headache disorders

I confirm that I have read this submission and believe that I have an appropriate level of expertise to confirm that it is of an acceptable scientific standard.

# Reviewer report: Reviewer response for version 1

**DOI:** 10.5256/f1000research.164311.r396524

**Published:** 2025-07-11

**Authors:** Lanfranco Pellesi

**Affiliations:** 1 University of Southern Denmark, Odense, Denmark

This is a well-structured, detailed, and clinically relevant study protocol describing a trial evaluating a novel, multimodal, therapist-independent biofeedback system (Cerebri) for migraine prevention. I have some comments:

**MAJOR COMMENTS**

- The protocol acknowledges the absence of blinding, but the implications are not adequately explored. I suggest expanding the implications in the Discussion section.
- Prior studies with the Cerebri prototype reported very low adherence in the second half of treatment, declining to 20% in week 9-12. Have you considered specific adherence enhancement strategies (e.g., automated reminders, motivational interviewing, in-app gamification, incentives)? Alternatively, have you considered doing sensitivity analyses excluding low-adherence participants?
- The sample size calculation is based on a moderate effect size (d = 0.4) derived from older meta-analyses of therapist-guided biofeedback. However, therapist-independent digital interventions have reported lower effect sizes (and higher variance). The effect size may be overestimated.
- How will missing data be handled statistically (e.g., multiple imputation, sensitivity analyses)?

**MINOR COMMENTS**

- Abstract: recheck verb tenses (the study is not yet done, right?) and add a short statement on key limitations (e.g., open-label design, adherence risks).
- Manuscript: several verb tenses are conjugated in the past, please recheck carefully.
- Methods: Include details on how participants will be instructed in device use remotely: are there standardized training materials?

Is the study design appropriate for the research question?

Yes

Is the rationale for, and objectives of, the study clearly described?

Yes

Are sufficient details of the methods provided to allow replication by others?

Yes

Are the datasets clearly presented in a useable and accessible format?

Not applicable

Reviewer Expertise:

Clinical trials in pain and headache disorders

I confirm that I have read this submission and believe that I have an appropriate level of expertise to confirm that it is of an acceptable scientific standard, however I have significant reservations, as outlined above.

# Reviewer report: Reviewer response for version 1

**DOI:** 10.5256/f1000research.164311.r314897

**Published:** 2024-08-30

**Authors:** Aynur Özge

**Affiliations:** 1 Department of Neurology, Mersin University, Mersin, Turkey

The manuscript titled "Biofeedback therapy using Cerebri for the prevention of migraine attacks in adults with episodic migraine (BioCer): a randomized, wait-list controlled trial – the study protocol" presents a study protocol aimed at investigating the efficacy and safety of a novel biofeedback device, Cerebri, in preventing migraine attacks in adults with episodic migraines. The device integrates three biofeedback modalities: heart rate variability (HRV), peripheral skin temperature, and electromyography (EMG), allowing users to engage in home-based biofeedback therapy without the need for healthcare personnel. The study is an open-label, randomized, waitlist-controlled, multicenter trial with a primary endpoint of evaluating the change in the mean number of migraine days from baseline to the last 28-day period of the treatment phase. The study is significant as it addresses the accessibility of biofeedback therapy, offering a therapist-independent, home-based solution for migraine management.

 My Comprehensive Comments are as follows; 

**Abstract:**

- The abstract is clear and concise, providing a good overview of the study's purpose, design, and significance. However, it would benefit from a brief mention of the study's limitations or challenges, such as the open-label design and the potential for low adherence, which is discussed in the manuscript.

**Methodology:**

- The methodology is thoroughly detailed, adhering to established clinical investigation standards. The inclusion of a baseline period as a screening phase is a strength, ensuring participants' eligibility is rigorously assessed.
- The open-label design, while necessary due to the nature of the intervention, introduces a potential bias that could be discussed more explicitly in the protocol. The lack of blinding is acknowledged, but further exploration of how this might affect the study's outcomes could enhance the robustness of the methodology section.
- The stratification and randomization process is well-explained, ensuring transparency in the study design.

**Results:**

- As this is a study protocol, the results section outlines the expected outcomes and statistical analyses planned. The use of a mixed logistic regression model is appropriate for the primary endpoint analysis, and the inclusion of both ITT and PP populations strengthens the study's rigor.
- The detailed plan for data management and statistical analysis, including handling protocol deviations, is a strong point of the manuscript.

**Discussion:**

- The discussion section appropriately situates the study within the broader context of migraine treatment research. The manuscript effectively highlights the innovation of combining multiple biofeedback modalities and the potential impact of this approach on migraine management.
- The discussion on the challenges of adherence to smartphone-based biofeedback systems is well-founded and crucial to understanding the study's potential limitations. However, the discussion could be expanded to include potential strategies for improving adherence, which could be beneficial for future implementations of similar interventions.

**Quality of Tables and Figures:**

- The tables and figures are clear and well-organized, providing necessary visual support to the study protocol. The flowchart (Figure 1) is particularly useful in illustrating the study design and participant flow.
- The screenshots of the biofeedback treatment (Figures 3 and 4) effectively demonstrate how the Cerebri system operates, which adds clarity for readers who may not be familiar with biofeedback technology.

This manuscript presents a well-designed study protocol for evaluating a novel biofeedback device in migraine prevention. While the manuscript is strong overall, I suggest a minor revision to address a few areas: the abstract could briefly mention study limitations, the methodology section could include a more explicit discussion of the potential biases introduced by the open-label design, and the discussion could offer suggestions for improving adherence to the biofeedback regimen. Additionally, while the tables and figures are well-prepared, ensuring that they are not overly complex or repetitive will be important as the study progresses and results are reported.

Is the study design appropriate for the research question?

Yes

Is the rationale for, and objectives of, the study clearly described?

Yes

Are sufficient details of the methods provided to allow replication by others?

Yes

Are the datasets clearly presented in a useable and accessible format?

Yes

Reviewer Expertise:

Neurology, Algology, and Clinical Neurophysiology specialist. headache expert. An active member of the Pediatric SIG of IHS for more than 20 years.

I confirm that I have read this submission and believe that I have an appropriate level of expertise to confirm that it is of an acceptable scientific standard.
